# Optimization design and application of library face recognition access control system based on improved PCA

**DOI:** 10.1371/journal.pone.0313415

**Published:** 2025-01-07

**Authors:** Na Lin, Yan Ding, Yulei Tan

**Affiliations:** 1 Library, Jilin Agricultural University, Changchun, China; 2 College of Artificial Intelligence Technology, Changchun Institute of Technology, Changchun, China; BMS Institute of Technology and Management, INDIA

## Abstract

The application of face recognition technology in Library Access Control System (LACS) has an important impact on improving the security and management efficiency of the library. However, the traditional face recognition methods have some limitations in the face of complex environmental conditions such as illumination and posture change. To solve this problem, an improved method combining the Aggregating Spatial Embeddings for Face Recognition (ASEF) algorithm and Principal Component Analysis (PCA) is proposed. The PCA algorithm is optimized by introducing beta prior and full probability Bayesian model. In addition, the research also integrates K-means Clustering Algorithm (KA) to further improve the accuracy and efficiency of face recognition. The experiment showed that the improved PCA method had an average recognition rate of 92.6%, an average recognition speed of 0.40s, and higher accuracy compared to other related methods, reaching 96%. In practical applications, the system quickly and accurately completes the identification of personnel entry and exit, and improves the efficiency and security of library access management.

## 1. Introduction

The Library Security System (LCS) and the LACS comprise a comprehensive management system that encompasses various security measures and access control policies to ensure the safety of individuals and property within the library. Among them, face recognition, as an important part of LCS/LACS, plays a key role in library access control management with its unique identity recognition capability [1]. Face recognition components typically consist of multiple modules that work together to achieve efficient and accurate face recognition. These modules include face detection, face alignment, feature extraction, and matching. Each module has its specific function and role, which together constitute a complete face recognition system [2,3]. However, traditional methods for face recognition have limitations when dealing with complex environmental conditions. Recognition accuracy and real-time performance are often difficult to guarantee, especially when there are large changes in lighting and pose differences [4]. The data that face recognition technology needs to process usually has a high dimension. Each facial image can be considered a high-dimensional data point, with each pixel value representing a feature. When dealing with large amounts of image data, this high dimension presents a host of problems. In addition, high-dimensional data is also more likely to lead to over-fitting problems, where the model performs well on training data but is less able to generalize on new data. Therefore, dimensionality reduction of data is required [5]. ASEF and PCA are combined in this study. The PCA is improved by introducing beta priors to construct a Bayesian model with full probability, thus achieving a more accurate face recognition effect. The improved PCA method is applied to the optimal design of the library Face Recognition Access Control System (FRACS).

The innovation of this research lies in the combination of a human eye location algorithm and PCA. This improves the PCA by introducing a beta before building a full-probability Bayesian model, resulting in a more accurate face recognition effect. This innovation not only solves the limitations of traditional face recognition methods in the face of complex environmental conditions but also provides a more efficient and secure solution for library access control management.

The contribution of the research lies in the improvement of the PCA algorithm, which improves the accuracy and robustness of face recognition by introducing advanced mathematical models and optimization techniques, especially when processing face images under complex environmental conditions. The improved PCA algorithm can significantly reduce the recognition delay and enhance the real-time response ability of the access control system while maintaining a high recognition rate. The study demonstrates the effectiveness of PCA in dimensionality reduction of high-dimensional data, improving the efficiency of data processing by reducing data dimensions while preserving critical information.

The article is divided into five sections. Section 1 is the introduction part, through the analysis of the background of library security management and the limitations of the existing research leads to the theme and content of this study. Section 2 is a literature review, which discusses and analyzes the current research status of library FRACS and PCA both domestically and internationally. Section 3 proposes an improved PCA and designs a library FRACS based on the ASEF algorithm to improve PCA. Section 4 verifies the effectiveness and performance of the algorithm through experiments. Section 5 is the conclusions, summarizing the research results.

## 2. Background and related works

The use of face recognition technology in LACS has become increasingly widespread, providing robust support for library security management and convenient services. However, the traditional face recognition methods still have some limitations in the face of complex environmental conditions such as illumination and posture, so the improvement and optimization of face recognition technology has been the focus of research. PCA has a wide range of applications in image processing, pattern recognition, and data analysis, which can help reduce the complexity of data, improve computational efficiency, and extract the most useful features for the problem.

Kar et al. demonstrated superior performance to state-of-the-art methods by effectively recognizing facial expressions with a hybrid feature descriptor and an improved classifier. The high efficiency and accuracy of this method in the facial expression recognition task were proved. However, the paper did not mention the performance of the method under complex environmental conditions, such as the change of illumination, attitude difference, and other factors that may affect the recognition effect. Studying the robustness of this method under varying environmental conditions and optimizing the feature descriptor and classifier could improve recognition accuracy [6]. Okokpujie et al. used 4-layer convolutional neural networks to build a face recognition system that is not affected by light, with high recognition accuracy and relatively few iterations. This showed the effectiveness of convolutional neural networks in illumination robust face recognition. While the method performed well under lighting conditions, the paper did not mention performance under other complex environments such as attitude changes, occlusion, etc. The article suggested exploring the method’s performance under a wider range of environmental conditions and improving recognition speed and accuracy [7]. Ayo et al. realized automatic face detection through geometric analysis and the YOLO algorithm with high accuracy and the ability to ignore non-face objects. This provided a basis for the construction of security analysis and face recognition systems. Although this method performed well in face detection, the paper did not mention subsequent face recognition performance [8]. Alsawwaf et al. proposed face recognition based on feature distance and position ratio of frontal images, and verified the performance of the proposed method by precision, error rate, and accuracy. The limitation of this article is that it has not been compared with other advanced methods, and its performance can be evaluated by comparing it with other advanced methods [9]. Kapse et al. used a variety of algorithms to build an efficient and reliable face recognition system, which is suitable for classroom attendance and other scenarios. The system was inexpensive and easy to install. The paper did not mention the performance of the method on large data sets or complex environments, nor did it discuss the scalability of the system. Future studies could study the performance of this method in large data sets and complex environments, as well as explore ways to improve the scalability and accuracy of the system [10]. Yeol et al. built a DLib-based face recognition visitor access system, which realized the functions of face scanning, recognition, real-time verification, and video storage. The system worked well in practical application but did not mention the performance of the method under a large number of concurrent accesses or complex environments. Additionally, how to optimize the system to improve processing speed and accuracy should be explored [11].

Cai et al. realized efficient fault monitoring by combining the dynamic recursive kernel PCA model with the variable Bayesian Gaussian mixture model. This method improved the accuracy of fault detection and identification, and reduced the false positive rate. Although this method performed well in fault monitoring, the paper did not mention its application in face recognition or other biometric technologies [12]. By combining PCA and the A* algorithm, Li et al. realized optimal monitoring and path planning of petrochemical processes. The effectiveness of this method was verified in practical application. In the future, how to apply this method in the field of face recognition, especially in processing high-dimensional data and optimizing feature extraction should be studied [13]. Pascual et al. proposed a supervised dimensionality reduction method called least square regression PCA, which is suitable for both classification and regression problems. The study examined the application of this method in face recognition, compared its performance with other dimensionality reduction methods (such as PCA, LDA, etc.), and explored the combination of other advanced technologies to improve face recognition accuracy [14]. Manoilov et al. built a nanosequencer based on PCA to improve sequencing accuracy. This provided a new idea for the research of sequencing technology. It was possible to explore how to optimize feature extraction and recognition performance [15]. Kanuboyina et al. proposed a PCA-based automatic classification of emotional states with improved recognition accuracy compared to support vector machines on both databases. This paper did not discuss how to deal with the recognition problem in a complex environment. In the future, how to apply this method in real-time should be studied [16].

In summary, PCA, as a commonly used data reduction and feature extraction method, has shown its strong application potential in many fields. Although the existing research has made remarkable progress in improving the recognition accuracy and processing speed, there are still some gaps and limitations. Firstly, the majority of existing studies concentrate on the ideal scenario in laboratory settings, with a paucity of research examining recognition performance in the context of complex environmental alterations. Secondly, although some studies try to improve recognition accuracy by fusing multi-modal information, these methods are often computationally expensive and difficult to meet the needs of real-time processing. Finally, although a variety of face recognition algorithms have been proposed, their adaptability and generalization ability in different application scenarios still need to be further verified. Especially in public places such as libraries, how to balance security and user convenience to develop more intelligent and user-friendly systems is a blank spot in the current research. The research aims to fill these gaps by proposing a library FRACS based on improved PCA to improve the accuracy and speed of recognition, while enhancing the robustness and practicality of the system.

## 3. Design and application of FRACS based on the improved PCA library

This study mainly focuses on improving the design of facial recognition algorithms based on the PCA technology. The focus of PCA improvement involves face image segmentation, optimization of facial feature merging algorithms, etc. The face recognition process mainly covers multiple aspects such as reading into the face database and distance function.

### 3.1 Facial recognition data dimensionality reduction and feature extraction based on the improved PCA

**Fig 1:**
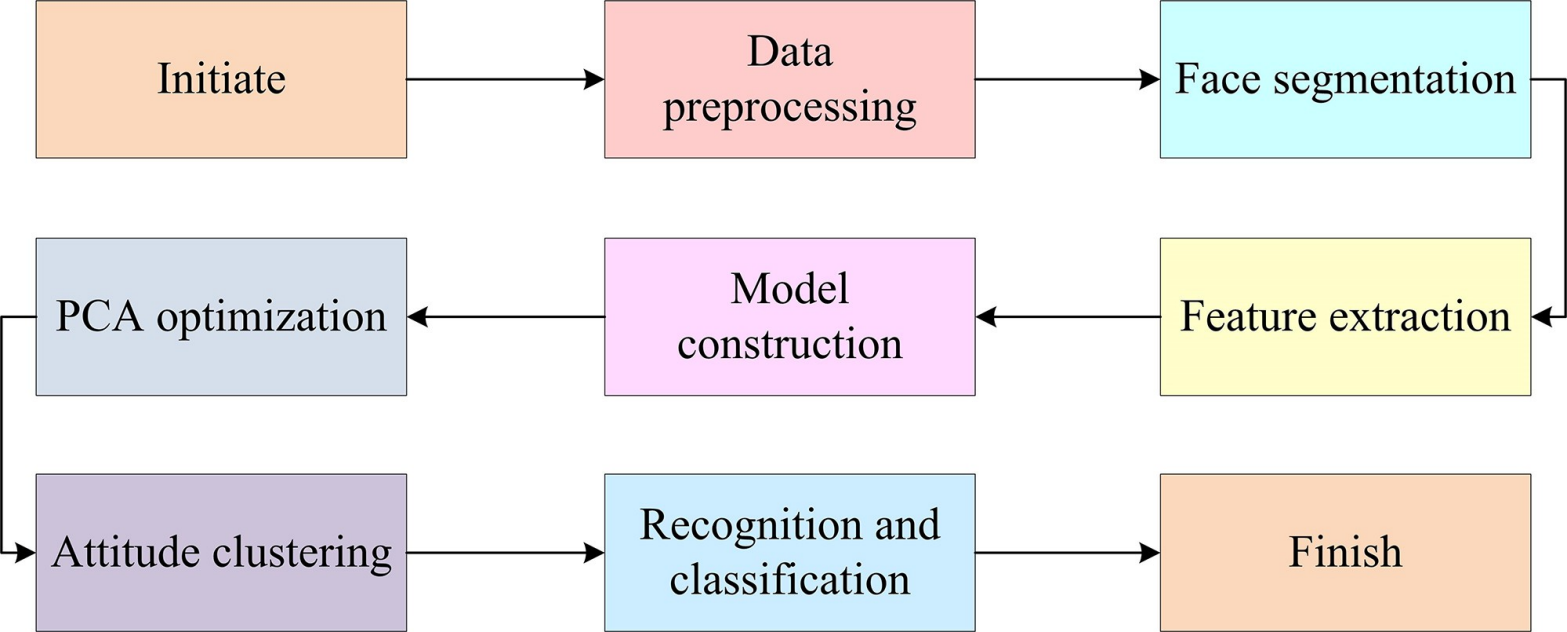
Flowchart of the face recognition system.

In this section, the study delves into an improved PCA-based face recognition technology aimed at improving the accuracy and robustness of recognition through advanced data reduction and feature extraction techniques. The research starts with the accurate segmentation of the input face image, which not only simplifies the recognition process but also helps to extract the key digital features in the image. Then, PCA is used to extract the features of the segmented images, which lays the foundation for the recognition process by retaining key information and reducing the data dimension. To further improve the performance of the recognition technology, beta prior is introduced and a full-probability Bayesian model is constructed, which improves the traditional PCA method. Face recognition technology is a biometric recognition method that employs computer vision to analyze face images and extract feature points, including eyes, nose, and mouth. These features are then compared with known face models to achieve accurate identity matching [17,18]. This process is the core of a face recognition system, and the detailed system flow involved is shown in Fig 1. The research and improvement method, which is based on a series of coherent technical steps, optimizes the face recognition process and enhances the stability and reliability of the system when dealing with complex environmental changes. This provides a solid technical foundation for the library FRACS.

As shown in Fig 1, face recognition mainly involves reading the input image, completing preprocessing according to requirements, and then segmenting the image after the preprocessing is completed. This can significantly reduce the difficulty of image recognition. There is a very close relationship between image recognition and image segmentation technology. After performing segmentation operations, the required digital features can be obtained, which can be well utilized. Segmentation is an essential aspect of image processing, and its accuracy is relevant to the effectiveness of image processing. This technology covers many types, including typical histogram correction and enhanced contour. The clarity of the image has a significant impact on the viewer’s senses. The segmentation operation is carried out combined with the relevant requirements of the research, which include the recognition and location of specific objects in the image, image editing, and synthesis. The purpose of the segmentation operation is to identify and extract the target region accurately. This requires the segmentation algorithm to have high precision and can avoid false segmentation or missing segmentation to obtain reliable results. During the segmentation process, it is necessary to first clarify the target area to ensure that the segmentation technology can be carried out in an orderly manner and meet the research needs. Next, feature extraction is carried out, usually requiring reading the features of the nose, eyes, and other parts. Then, the PCA method is used to study them and compare them with the information existing in the database. Finally, it compares the output of the results. PCA transforms the raw data into a set of orthogonal principal components through linear transformation, where each principal component is a linear combination of the raw data. These principal components are sorted according to the size of variance, thus preserving as much important information as possible from the original data. The core idea of PCA is to map high-dimensional data to a low-dimensional space while preserving the information of the original data to the greatest extent possible. Specifically, PCA determines the direction of the principal components by calculating the eigenvalues and eigenvectors of the covariance matrix, and selects the most important principal components on the ground of the size of the eigenvalues. After mapping data to low dimensional space, PCA can be used for tasks such as data visualization, classification, clustering, and noise reduction. Feature face algorithms mainly extract target features through PCA, and in general, these methods can be regarded as a type of facial image description method. Its core principle is to use transformations as the core to extract valuable facial features and obtain the required eigenvalues. Afterward, the eigenvalues are chosen to construct a lower dimensional orthogonal basis space. In addition, the face can be projected into a lower dimensional space to obtain the feature face space. After performing operations on the recognized image, it is possible to determine the distance between the feature face space and the training image. If the distance is relatively close, it indicates that the similarity between the two is relatively high, which enables efficient facial recognition. In addition, the dimensionality of the projected space is usually low, and the feature face space retains the effective information of the initial image [19]. Its assumption is that the original image is represented by *p* indicators, and then the initial variables are represented by *X*_1_,*X*_2_,…,*X*_*P*_. Each variable is mainly used to represent a one-dimensional vector. At this point, *p* variables form a *p*-dimensional vector, and *X* = (*X*_1_,*X*_2_,…,*X*_*P*_)′. Afterward, the mean of *X* can be represented by *μ*, mainly representing the covariance matrix corresponding to *X*. According to the requirements, a linear transformation is performed on *X*, and the comprehensive variable is generally represented by *Y*. Afterward, this variable is specifically shown in Eq (1).


$$\{\begin{array}{c} Y_{1}=\mu_{11}X_{1}+\mu_{12}X_{2}+\ldots +\mu_{1P}X_{P}\\ Y_{2}=\mu_{21}X_{1}+\mu_{22}X_{2}+\ldots +\mu_{2P}X_{P}\\ \ldots \\ Y_{P}=\mu_{P1}X_{1}+\mu_{P2}X_{2}+\ldots +\mu_{PP}X_{P} \end{array}$$ (1)

In Eq (1), *X* represents the raw data matrix, where each column can represent a sample, and each row can represent a feature or variable. *p* represents the number of features or variables, i.e. the dimensions of the original data. *μ* represents the mean vector, and for each row of *X*, the corresponding element in *μ* is the mean of that row. *Y* represents the new variable after the linear transformation and the principal component. After linear combination, a comprehensive variable with diversity can be obtained. Therefore, to optimize the reconstruction effect, the variance of *Y*_*i*_ = *μ*_*i*_*X* should be maximized, and each *Y*_*i*_ should have relatively high independence. The constant *c* is specifically shown in Eq (2).


$$\mathrm{var}(cu_{i}^{\prime }X)=cu_{i}^{\prime }{\sum_{i=1}^{p}u_{i}cu^{\prime }}_{i}\sum_{i=1}^{p}c^{2}u_{i}$$ (2)

**Fig 2:**
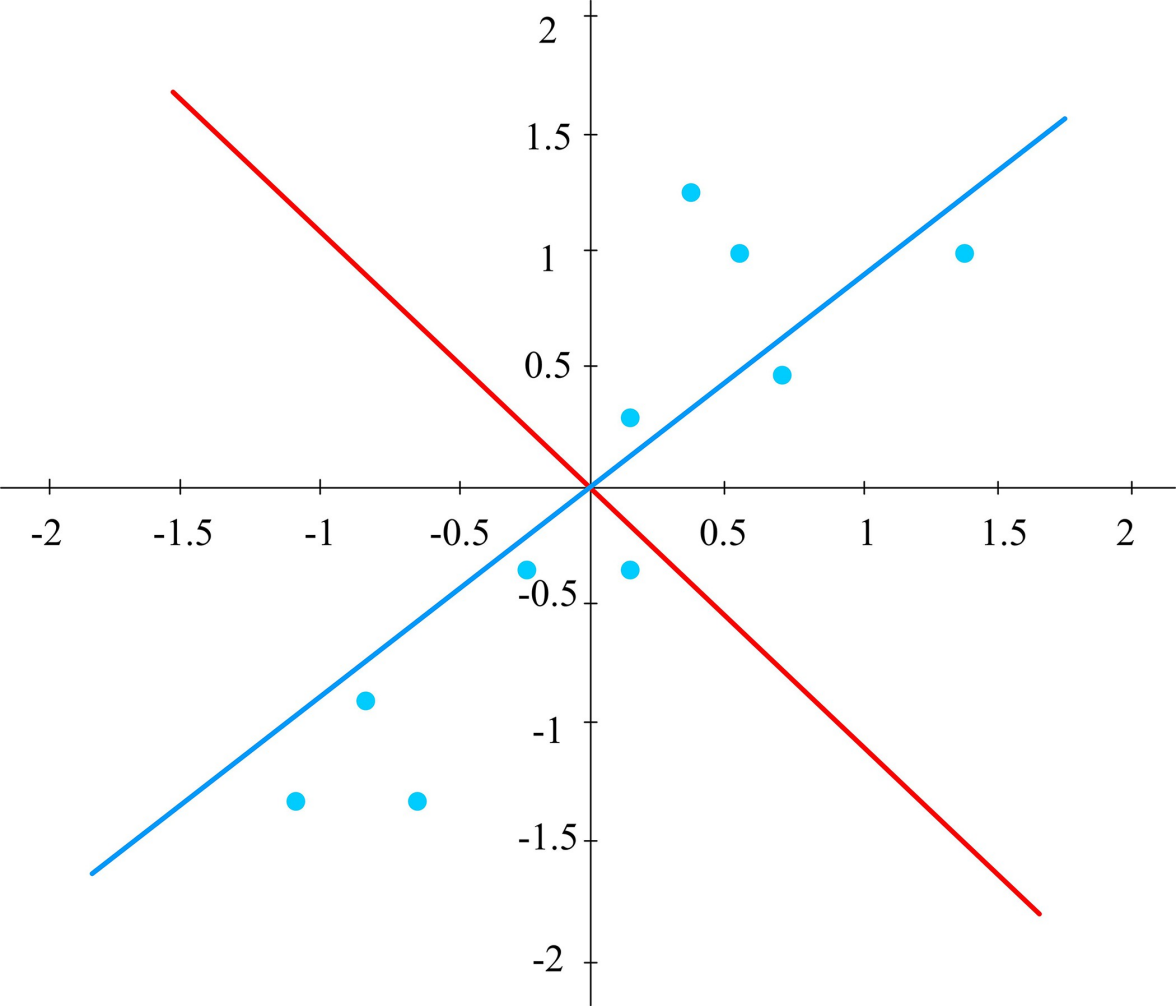
Two-dimensional data projection diagram.

In Eq (2), *c* represents a coefficient vector. *u*_*i*_ represents the direction of the *i*-th principal component, and $u_{i}^{\prime }$ is the transpose of *u*_*i*_. *X* is the original data matrix, and Σ is the sum over all the features. The proportion of each variable in the total variance is constantly decreasing. In reality, components with prominent variances are usually selected to meet the requirements of dimensionality reduction. Compared to the original vector, the new vector corresponds to a relatively small number of columns and typically only selects essential data items. This can refer to the original data and effectively reduce the difficulty of data analysis, and filter out many worthless data [20]. The basic principle of PCA technology is shown in Fig 2.

In Fig 2, the two axes mainly refer to two-dimensional data, while the blue dots specifically refer to two-dimensional data points, the blue line is the initial coordinates, and the red line is the coordinates after the conversion. If the data of a certain axis is projected onto the space of other axes, then it can be dimensionally reduced. Applying this method could significantly improve computational efficiency while maintaining a relatively low level of difficulty. The principle of PCA can assume that it covers *N* images, each with a corresponding dimension of *M*. Afterward, a matrix *B* is constructed, which is composed of *N* images. At this point, each row vector refers to the image. Therefore, the specification of matrix *B* is 5*4. The corresponding schematic diagram is shown in Eq (3).


$$B=[\begin{array}{cccc} b_{0,0} & b_{0,1} & b_{0,2} & b_{0,3}\\ b_{1,0} & b_{1,1} & b_{1,2} & b_{1,3}\\ b_{2,0} & b_{2,1} & b_{2,2} & b_{2,3}\\ b_{3,0} & b_{3,1} & b_{3,2} & b_{3,3}\\ b_{4,0} & b_{4,1} & b_{4,2} & b_{4,3} \end{array}]$$ (3)

In Eq (3), *B* represents the data matrix, where each row is an image sample, and each column is a feature. *b*_*m*,*n*_ is the sample in the matrix, where *m* is the number of samples and *n* is the number of features. The mean values of each column are analyzed in matrix B, and the corresponding formula is shown in Eq (4).


$$u(m)=\frac{1}{n}\sum_{n=1}^{N}X(m,n)$$ (4)

In Eq (4), *u*(*m*) represents the mean of the *m*-th column in matrix *B*, that is, the *m*-th feature. *n* is the number of columns of matrix *B*, which is the number of features. *X*(*m*,*n*) is the element of row *m* and column *n* in matrix *B*, that is, the NTH eigenvalue of the *m*-th sample. The expression of matrix *E* is shown in Eq (5).


E=B−U (5)

In Eq (5), *B* represents the original data matrix, *U* represents the mean value matrix, and *E* represents the data matrix after centralization. The composition of *U* is specifically shown in Eq (6).


$$U={\left(\begin{array}{ccc} u(1) & \ldots & u(1)\\ \vdots & \ldots & \vdots \\ u(M) & \ldots & u(M) \end{array}\right)}_{M\times N}$$ (6)

In Eq (6), *u*(*M*) is the mean of column *M* of matrix *B*. The covariant matrix *C* is shown in Eq (7).


$$C=\frac{1}{N}\sum_{i=1}^{N}EE^{T}$$ (7)

In Eq (7), *C* is the covariance between random variables. The specification of the covariant matrix *C* is *M*×*N*. For the covariant matrix *C*, the following relationship is satisfied, as shown in Eq (8).


|λI−C|=0 (8)

In Eq (8), *λ* is the eigenvalue, which is a scalar, with *λ* = *λ*_1_,*λ*_2_,…,*λ*_*m*_ and *λ*_1_≥*λ*_2_≥…≥*λ*_*m*_. *I* is the identity matrix. A square matrix whose diagonal elements are all ones and the rest are all zeros. A square matrix represents the covariance between different random variables. The eigenvector matrix is analyzed, and the calculation process is shown in Eq (9).


$$V^{-1}CV=0$$ (9)

In Eq (9), *V* is the eigenvector matrix, and *V*^−1^ is the inverse of *V*. The expression of the feature vector *D* is specifically shown in Eq (10).


$$D={\left[\begin{array}{ccccc} \lambda_{1} & 0 & 0 & 0 & 0\\ 0 & \lambda_{2} & 0 & 0 & 0\\ \vdots & 0 & \ddots & 0 & 0\\ 0 & 0 & 0 & \lambda_{M-1} & 0\\ 0 & 0 & 0 & 0 & \lambda_{M} \end{array}\right]}_{M\times N}$$ (10)

**Fig 3:**
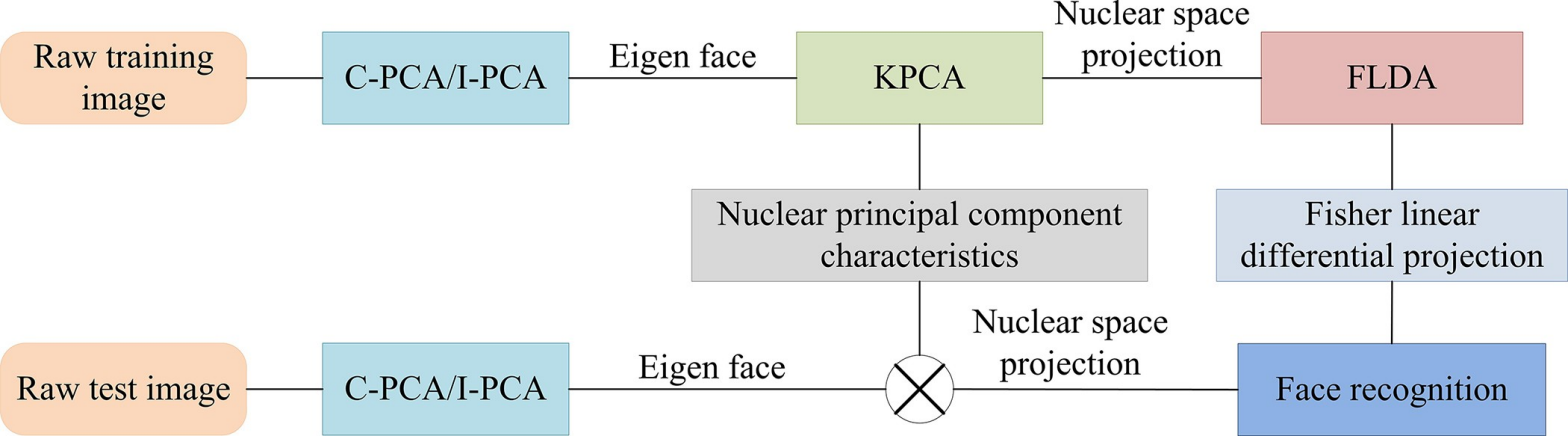
Face recognition algorithm flow based on the PCA.

In Eq (10), matrix *V* mainly represents the eigenvector matrix, and the eigenvalues corresponding to row *m* are *λ*_*m*_. This study selects the most applicable feature vector as the base, and in addition, the first *W* feature vectors can be selected, which must meet the following criteria *W*≤*M*. It projects the original *N* images onto the specified base, and then the first *W* projection values can be taken. Finally, it saves the feature parameters of each part to the database. The process of conventional PCA is shown in Fig 3.

In Fig 3, the steps of conventional PCA are relatively complex, with typical examples including database establishment, facial recognition, etc. Through this diagram, the working process can also be clarified, and certain areas need to be optimized. In applying traditional algorithms, the main focus is to select important feature points from the entire face, which are not unique to the face, thus hindering the optimization of the face recognition rate. Extracting data is mainly judged based on the distance between feature vectors, rather than setting specific weights based on the importance of feature points. In this way, each feature point is usually treated equally, and in specific scenarios, the distance difference between the feature vectors is too small, which affects the recognition results. Therefore, it is necessary to improve PCA by introducing a beta before constructing a full probability Bayesian model. The expression of the improved PCA model is shown in Eq (11).


$$\{\begin{array}{c} s=UZU^{T}s+\varepsilon \\ z_{k}∼Bernoulli(\pi_{k})\\ \pi_{k}∼Beta(a_{0},b_{0})\\ \varepsilon ∼N(0,\gamma^{-1}I) \end{array}$$ (11)

In Eq (11), *z*_*k*_ is the *k*-th diagonal element in the diagonal matrix *Z*, and *z*_*k*_∈{0,1}, therefore it can be considered that *z*_*k*_ follows the Bernoulli distribution, i.e. *z*_*k*_∼*Bernoulli*(*π*_*k*_). *π*_*k*_ represents the probability of *z*_*k*_ = 1. To construct a complete Bayesian architecture, a beta prior is introduced to *π*_*k*_, where *π*_*k*_∼*Beta*(*a*_0_,*b*_0_). *a*_0_,*b*_0_ are hyper-parameters. *ε* is the noise component, which is usually assumed to be independent of the signal component and follows a 0 mean Gaussian distribution, i.e. *ε*∼*N*(0,*γ*^−1^*I*). This improved PCA can automatically determine the number of principal components. The use of PCA in the study not only reduces the dimensions of the data but also helps to identify and eliminate noise and redundancy in the data, thus improving the training efficiency and classification performance of the model. The differences between different individuals can be identified more clearly through the data transformed by PCA, which provides a more accurate feature representation for subsequent classification tasks. The improved PCA makes the dimensionally reduced data more suitable for face recognition tasks.

### 3.2 Face recognition algorithm on the ground of ASEF and KA

**Fig 4:**
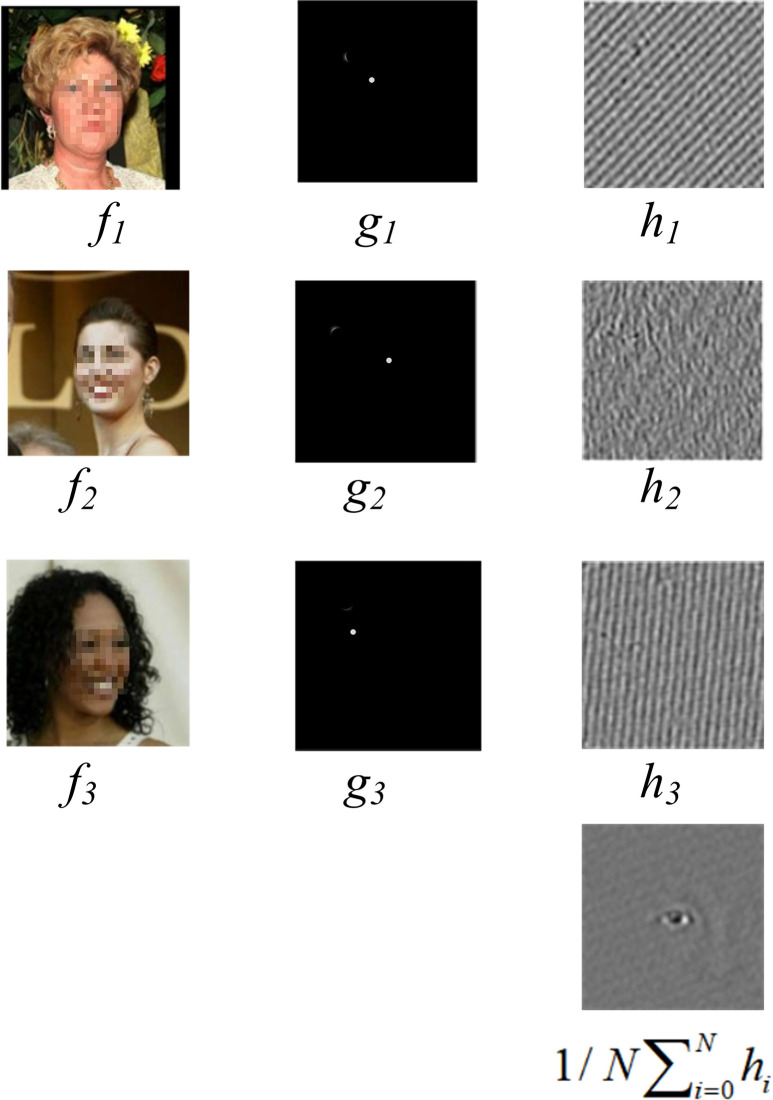
Construction process of ASEF filter.

In this section, an advanced face recognition algorithm that integrates the ASEF algorithm and KA is studied in detail, which is designed to significantly improve the accuracy and efficiency of face recognition. The research commences with the preprocessing of the original face image, wherein filtering technology is employed to enhance the image features and reduce the noise. This serves to establish a robust foundation for the subsequent processing. Subsequently, the ASEF algorithm is employed to precisely extract data regarding the position of the human eye, a crucial step in accurately capturing the essential characteristics of the face. To ascertain the position of the human eyes, the research further adopts the use of KA, which is an effective method of classifying images according to the pose of the face. This ensures that recognition is more accurate within the same pose category. On this basis, combined with the face shape and expression features, the Adaboost algorithm is used for the final face recognition and classification, which further improves the overall recognition performance of the system. In addition, PCA is improved to make it more suitable for data reduction and feature extraction in face recognition. The enhanced PCA technology, when integrated with image filtering techniques, not only streamlines the pre-processing of facial images but also enhances the precision of extracting human eye position data [21]. The construction process of the ASEF filter is shown in Fig 4.

In Fig 4, the face photos are from the CelebA Face Dataset, which is an open source dataset. Dataset link: http://m6z.cn/60EW0n. The basic idea of the filtering operation is to use the value of adjacent pixels to determine the output of the current pixel and realize the image preprocessing through sliding and convolution operations. The filter with a smooth denoising function is mainly used to reduce the noise in the image and improve the image quality. The filter with edge detection function is mainly used to highlight the edge information in the image, which is helpful for the subsequent image analysis and recognition. The key steps in building an ASEF filter include processing input samples, generating composite output results, and constructing a frequency domain filter. Specifically, the input sample needs to be preprocessed first to extract the information related to the human eye position. Then, based on this information, one or more filters are constructed in the frequency domain. Finally, these filters are averaged and the average filter is obtained. This process can effectively use the feature information of the input sample to improve the accuracy and stability of human eye positioning. The idea of ASEF algorithm is for learning a filter *h* and then performing convolution operation with image *f*_*i*_, as shown in Eq (12).


$$g_{i}=f_{i}\times h$$ (12)

**Fig 5:**
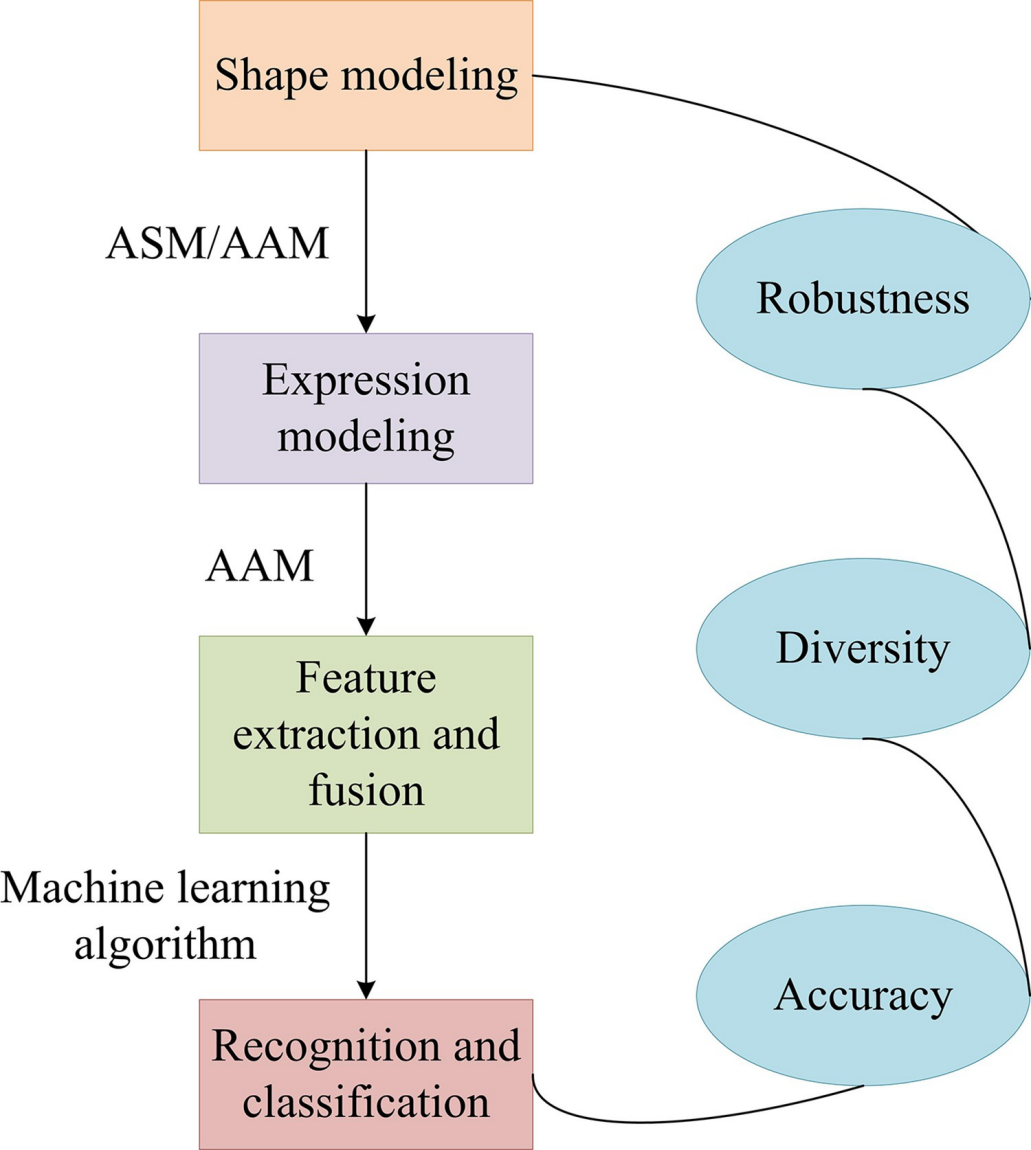
Main steps and advantages of ASEF algorithm.

The ASEF algorithm fully utilizes the information of facial shape and expression, and can better cope with the impact of lighting changes, posture changes, and other factors on facial recognition [22]. The main steps and advantages of the ASEF algorithm are shown in Fig 5.

In Fig 5, the algorithm first uses a set of labeled face images to build a Shape Model, utilizing either the Active Shape Model or the Active Appearance Model. Then, based on the face shape, the expression model is further established through the marked expression samples. In the analysis of the recognized face images, the shape and expression features are extracted. The former is determined by the position coordinates of key points, and the latter is obtained by analyzing the texture and dynamic changes of expressions. These features are then fused to form a comprehensive feature representation, which can improve the robustness of the algorithm under conditions such as illumination and attitude changes. Next, the study uses machine learning algorithms to process these comprehensive features to achieve high-precision face recognition and classification. Although the ASEF algorithm has performed well in improving recognition accuracy and adaptability, it also faces some challenges, such as its reliance on high-quality labeled data and high computational complexity. Therefore, to achieve optimal performance, it is necessary to carefully select training samples, adjust parameters, and perform appropriate preprocessing and optimization. Furthermore, the research employs the use of KA, which is capable of effectively clustering data by collecting human eye distance information and applying KA, thus further enhancing the overall performance of the face recognition system. The KA is an unsupervised learning algorithm that directly puts unlabeled datasets into the algorithm to automatically generate *K* clustering centers and complete clustering operations. When performing algorithm operations, the first step is to randomly select *K* clustering centers from the image or information, and then classify the points closest to the point into that category through clustering operations. Afterward, it repeatedly recalculates the clustering center to determine the convergence position of the function. The specific algorithm inference is shown in Eq (13).


$$F=\sum_{i=1}^{k}\sum_{x\in C_{i}}^{n}{\left\|x-u_{i}\right\|}_{2}^{2}$$ (13)

In Eq (13), *F* is the minimum squared error of cluster partitioning obtained from sample clustering. The expression of *u*_*i*_ is shown in Eq (14).


$$u_{i}=\frac{1}{\left|C_{i}\right|}\sum_{x\in {C_{i}}^{x}}^{n}x$$ (14)

In Eq (14), *u*_*i*_ is the mean vector of cluster *C*_*i*_. The smaller the *E*, the closer the samples within the cluster are around the cluster center. The most important method used in KA is the algorithm for finding the center of the point group, which is the Euclidean distance. The n-dimensional data are taken as an example, which is shown in Eq (15).


$$dist(X,Y)=\sqrt{\sum_{i=1}^{n}{\left(x_{i}-y_{i}\right)}^{2}}$$ (15)

**Fig 6:**
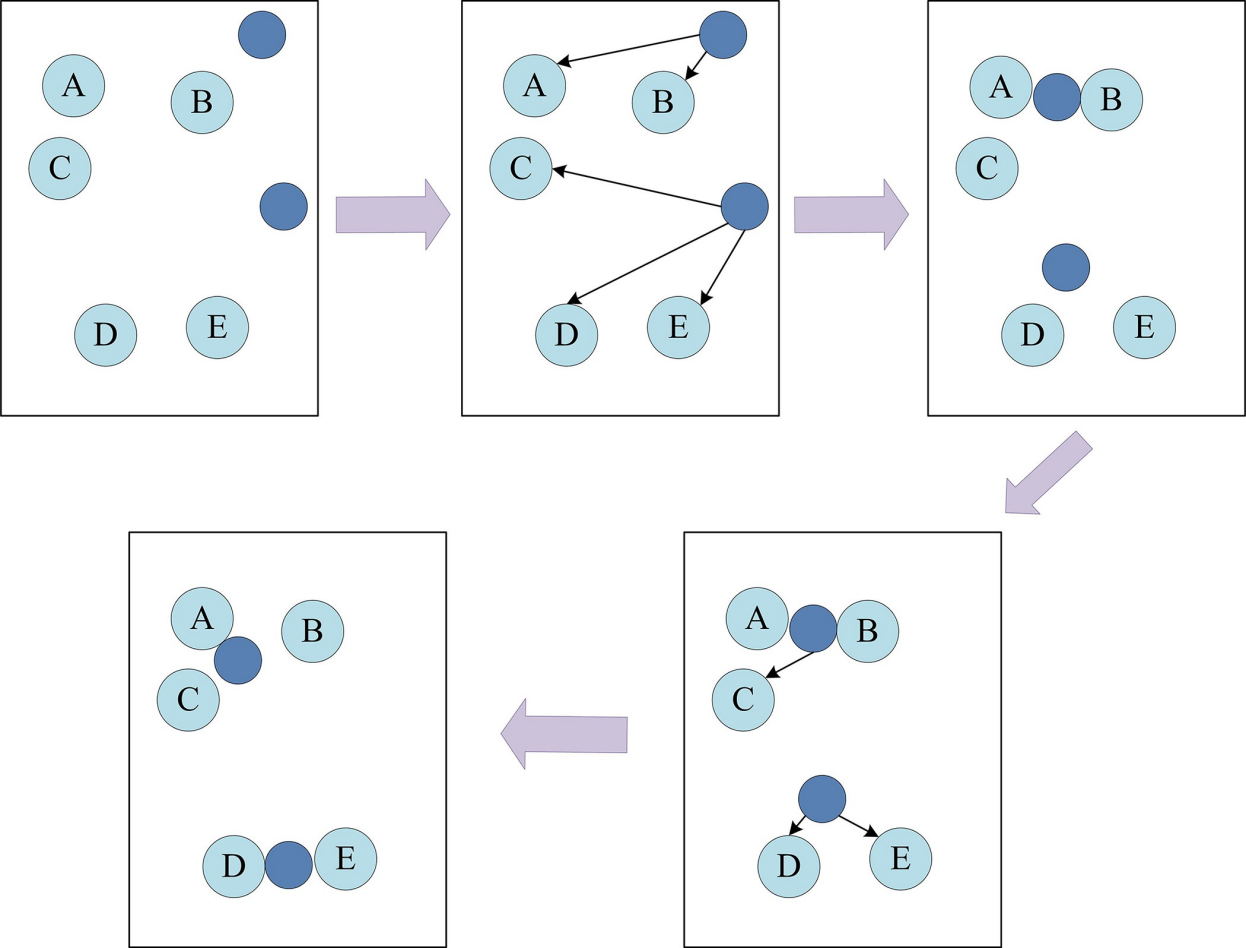
A simple example of the KA.

A simple example of the KA at *K* = 2 is shown in Fig 6.

Fig 6 shows an example of a simple KA for clustering face images in a face recognition system. In this example, supposing there are multiple data points in a two-dimensional space that represent the feature vectors of different facial images. Initially, two cluster centers are randomly selected, and each cluster center attracts surrounding points based on its proximity to the data point, forming two clusters. As the algorithm iterates, the position of the cluster center is adjusted according to the average position of all points in the cluster. This process is repeated until some termination condition is met, such as a maximum number of iterations or a change in the cluster center less than a set threshold. Information entropy is employed to quantify the degree of hybridization in clustering results. A higher information entropy indicates a greater number of facial categories present within each cluster, reflecting a higher degree of hybridization. Conversely, the degree of hybridization in clustering is low. The formula is shown in Eq (16).


$$Entropy=-\sum_{k=1}^{K}p_{mn}\;\log_{2}\;p_{mn}$$ (16)

**Fig 7:**
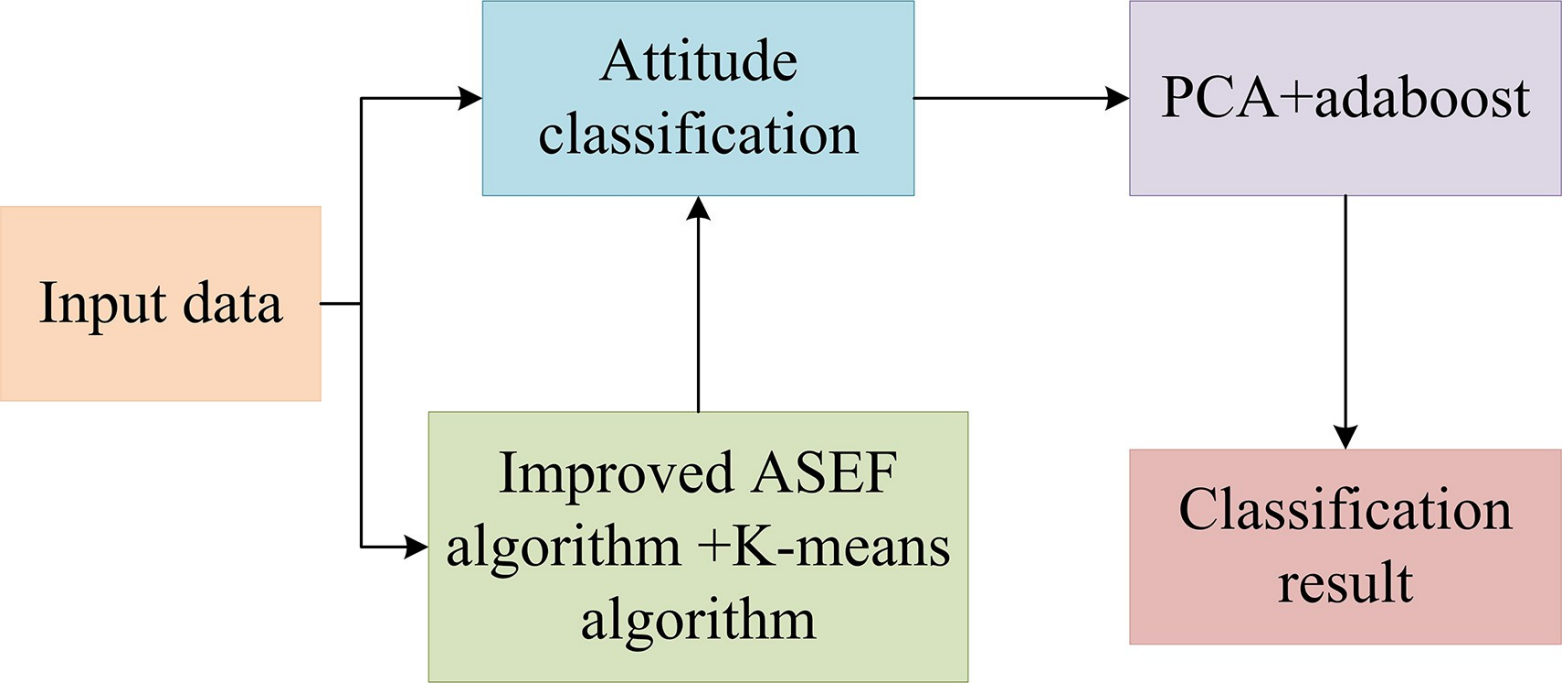
Face recognition algorithm flow.

In Eq (16), *P*_*mn*_ = *c*_*mn*_/*c*_*m*_, and *c*_*mn*_ is the number of category *n* in the *m*-th cluster. *c*_*m*_ serves as the total of samples in the *m*-th cluster. The improved ASEF and KA methods first perform eye localization, and then perform KA based on the distance between the left and right eyes to achieve the effect of facial pose classification. Next, it performs identity recognition within the same pose dataset using the PCA feature extraction method, and the classifier uses the Adaboost algorithm. Adaboost algorithm iteratively combines several weak classifiers into a strong classifier to improve the accuracy and robustness of classification. The main concept is to train multiple weak classifiers on the same training set and assign varying weights based on their performance. These weak classifiers are then combined to form a strong classifier. In the process of operation, the Adaboost algorithm first assigns initial weights to the training samples and then trains the weak classifier in an iterative way. In each iteration, the algorithm trains a weak classifier based on the current sample weight and calculates its error rate. A classifier with a low error rate gets a higher weight, while a classifier with a high error rate gets a lower weight. Then, the algorithm updates the sample weights according to the error rate of the classifier, so that the misclassified samples get higher weights in subsequent iterations. PCA is responsible for extracting effective feature representations, while the Adaboost algorithm uses these features for classification and recognition. By using the features extracted by PCA and the strong classification ability of the Adaboost algorithm, the identification can be effectively carried out, and the performance and security of the system can be improved. The specific process is shown in Fig 7.

In Fig 7, numerous facial images are first collected and artificial landmark annotations are obtained. Then, the KA is applied to classify similar facial feature patterns. By encoding facial landmark positions as feature descriptors, a feature space is created to represent different facial expressions and postures. Next, it utilizes the constructed dataset to train the facial feature localization model. By using trained models, facial landmarks can be accurately located and detected in real-time scenes. In addition, the application of KA in facial feature localization also brings some advantages. It can generate labeled training data automatically, which reduces the need for manual labor and improves the efficiency of the localization process. In addition, the proposed method can also adapt to various facial appearances and postures, making it suitable for various applications in the field of facial analysis and recognition. The access control model developed in this study aims to create a secure and user-friendly system by ensuring that only authorized users can access sensitive resources through multi-level authentication. The design of the model starts from the accurate identification of the user’s identity. On this basis, the system adopts an advanced encryption algorithm to protect the user’s data and the setting of access rights. To improve the robustness of the system, an exception detection mechanism is introduced, which can monitor the access behavior in real-time and detect and respond to potential security threats in time. In terms of user interface, the focus is on intuition and ease of use, ensuring that users can easily authenticate and access requests. At the same time, it also provides detailed user guides and help documents, so that users can quickly understand the working principle and operation method of the system. By providing clear audit logs and access records, users and administrators can track and review all access activity. Finally, the model is designed with ethical and privacy considerations in mind, ensuring that all user data processing complies with relevant data protection legislation and taking appropriate measures to protect users’ privacy rights.

## 4. Performance testing of improved PCA library facial recognition access control system

To test and improve the performance of the PCA library FRACS, this study compares the accuracy, recall rate, recognition effect, recognition speed and rate, average confusion matrix, and positioning error of PCA and related algorithms. Finally, the performance of different facial recognition algorithms and the effectiveness of different clustering methods are compared.

### 4.1 Performance testing for improving PCA facial recognition

**Fig 8:**
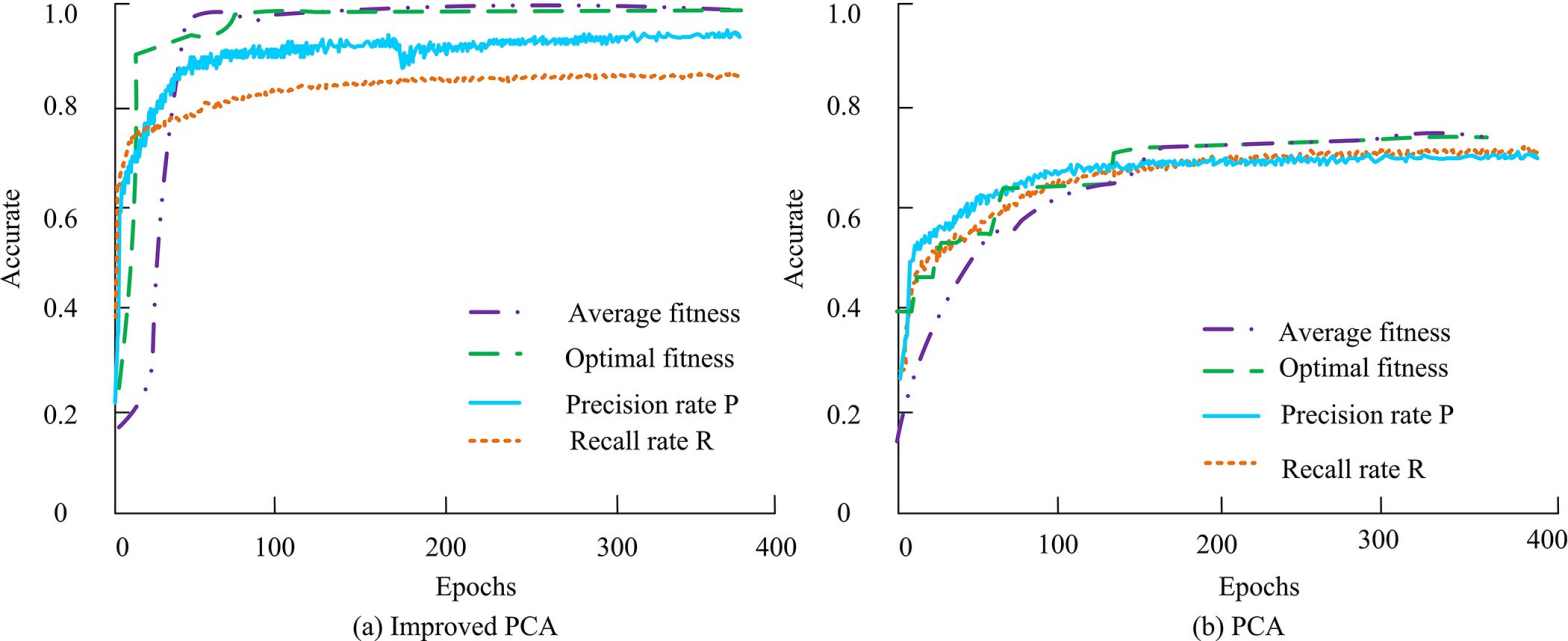
Precision and recall of the improved PCA model and the PCA model.

The face test set adopts the LFW dataset, with a population of 20–50 people and 2–19 images per person. The sample size increases as the number of categories increases, resulting in a total of 400 images. The LFW dataset is a widely used open source facial recognition database that contains photos of celebrities collected from the Internet. The LFW dataset is open-source. Researchers and developers are free to access and use this dataset for face detection, recognition, and other computer vision tasks. This dataset is only used in the experimental part of this study to verify the validity of the face detection technique proposed in this study. To test the performance of the improved PCA library FRACS, the accuracy, recall, and fitness of the improved PCA are compared and analyzed with those without improvement, as shown in Fig 8.

Fig 8A shows the accuracy and recall of the ASEF algorithm in improving PCA. It indicates that the accuracy index is relatively high, ultimately stabilizing above 0.9, and the recall index is also above 0.8. Fig 8B is the accuracy and recall rate of conventional PCA, with accuracy and recall indicators ranging from 0.7 to 0.8. This indicates that the improved PCA has a higher detection accuracy than before. To improve the accuracy of the PCA library’s FRACS, standardized testing of the system can be carried out through the ORL facial database. The study selects the first 5 images of all test individuals as the pre-stored database, and the last 5 images as the face database to be recognized. Afterward, it adjusts the dimensionality of the feature face space, allowing for standardized testing of recognition performance. This study selects 40 testers and conducts experiments with the entire group, as well as with the top 20 and top 10 testers. Afterward, the dimensionality of their feature spaces is adjusted, and the specific results obtained are shown in Table 1.

**Table 1:** Spatial dimension recognition rate and recognition time of 40 subjects.

Forty test subjects			The top 20 testers			The top 10 testers		
Feature face space dimension	Recognition rate (%)	Recognition time (s)	Feature face space dimension	Recognition rate (%)	Recognition time (s)	Feature face space dimension	Recognition rate (%)	Recognition time (s)
10	88	0.36	10	89	0.22	10	86	0.33
20	92	0.38	20	90	0.35	20	90	0.48
30	94	0.41	30	92	0.38	30	93	0.49
40	95	0.42	40	95	0.29	40	91	0.42
60	94	0.45	60	93	0.41	60	92	0.47

**Fig 9:**
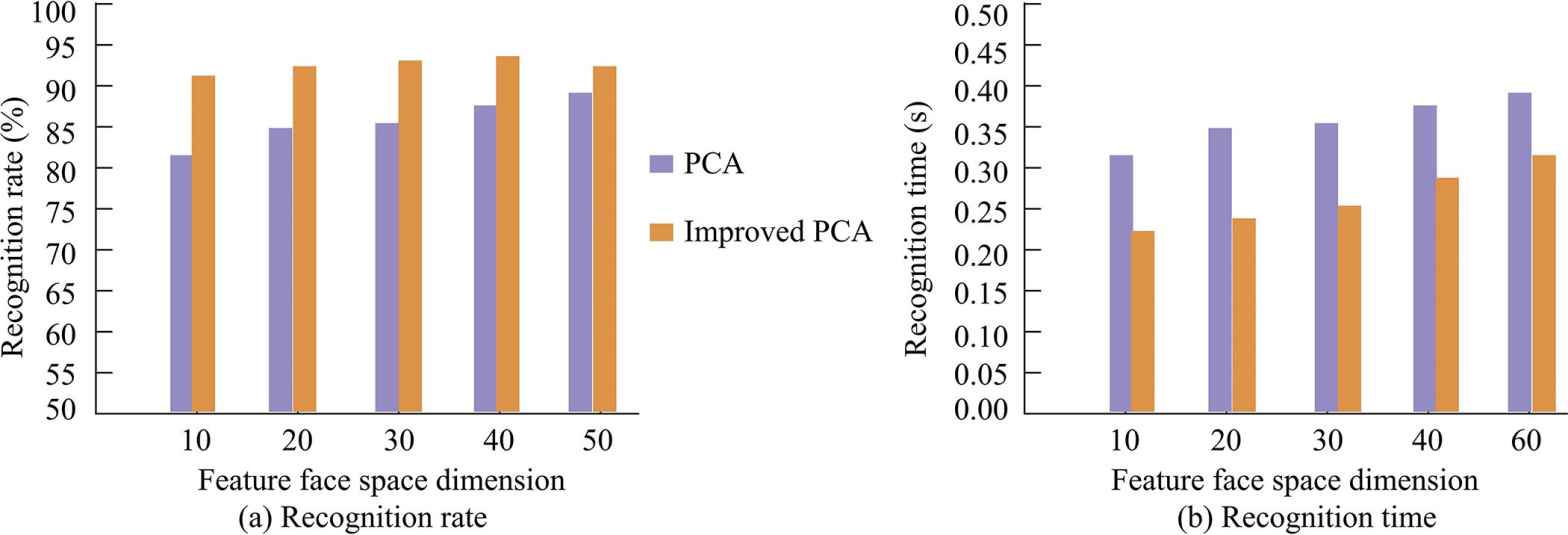
Recognition speed and recognition rate histogram.

In Table 1, the improved PCA library FARACS achieves an average recognition rate of 92.6% and an average recognition speed of 0.40 seconds. This indicates that the library’s FRACS has a high recognition rate and fast recognition speed. To provide a more intuitive and clear observation of the recognition speed and rate of the improved PCA library FRACS, a comparison is conducted between the improved PCA library FRACS and the conventional PCA library FRACS. The bar graph of recognition speed and recognition rate is shown in Fig 9.

**Fig 10:**
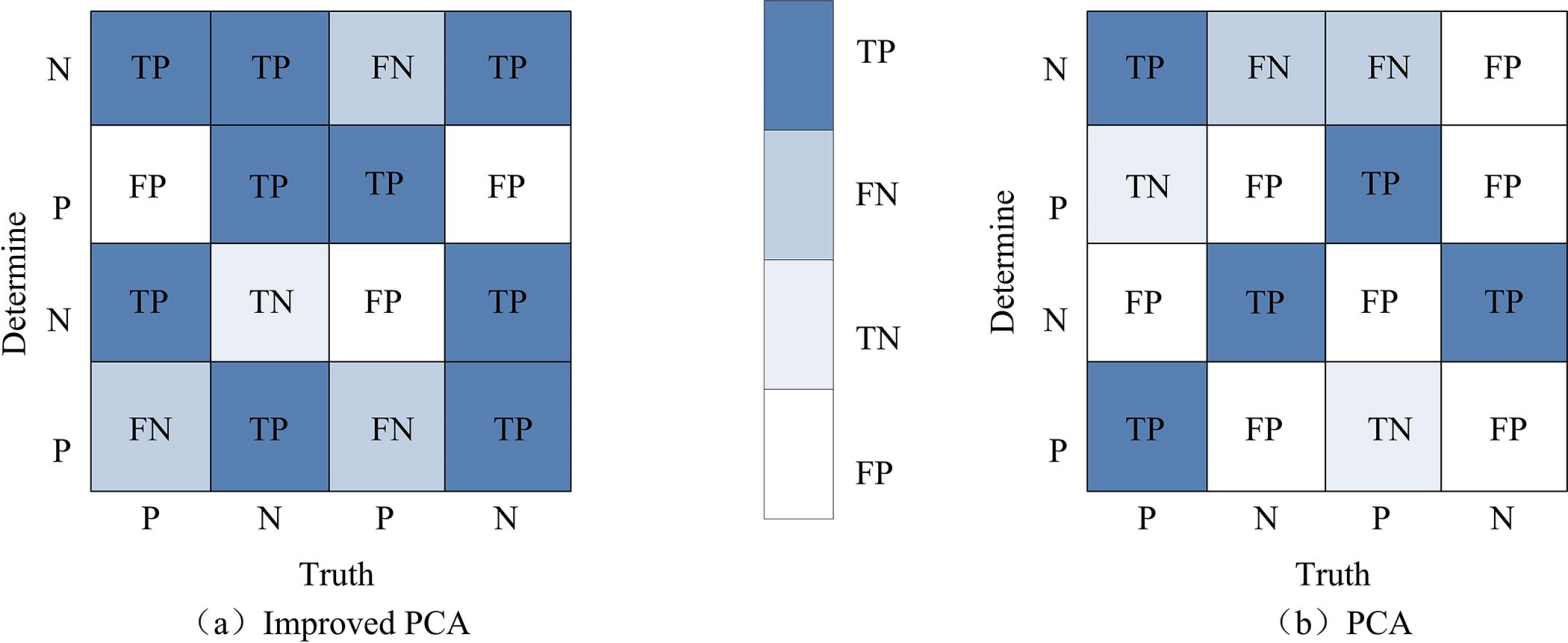
The average confusion matrix of PCA and the improved PCA.

Fig 9A and 9B represents the recognition rate and recognition speed under different feature face spatial dimensions. The recognition rate and speed of the improved PCA is higher than before, about 10% higher and 1 second faster. This indicates that the improved PCA has higher detection accuracy and better performance. To better demonstrate the recognition effect of the improved PCA library FRACS, this study introduces a multi-recognition confusion matrix to further quantitatively analyze the recognition effect. The average confusion matrix results of the improved PCA and conventional PCA are shown in Fig 10.

In Fig 10A is the confusion matrix of the improved PCA, and 10b is the confusion matrix of the conventional PCA. The improved PCA has identified more positive cases than the conventional PCA, with 13 actual positive cases compared to 7 in the conventional PCA. This indicates that the recognition accuracy of improved PCA is higher than that of conventional PCA. This study considers multiple poses and selects 20 samples to analyze the positioning error of the ASEF algorithm in improving PCA under different angles and lighting conditions, as shown in Table 2.

**Table 2:** ASEF algorithm improving the localization error of PCA algorithm.

Angle classification	Positioning error	Light condition	Positioning error
-90	3.6	1–5 strength mix	1.4
-60	2.8	1–10 strength mix	1.3
-30	2.6	1–20 strength mix	0.7
0	0.8	1 strength	2.9
30	1.4	20 strength	2.8
60	2.1	Single illuminance, average error	0.7
90	3.2	/	/

As evidenced by the experimental results presented in Table 2, a reduction in angle, or a closer positioning of the face to the front, is associated with a diminished positioning error. This observation suggests that the algorithm demonstrates enhanced accuracy in processing face images that are oriented toward the front or close to it. On the contrary, when the Angle increases and the face gradually turns to the side, the difficulty of positioning increases, resulting in greater errors. Under extreme lighting conditions, the positioning error of ASEF-improved PCA is relatively high regardless of the worst or best lighting. This is because the lighting conditions have a significant impact on the contrast of the image and the clarity of the features, thus affecting the accuracy of the positioning. However, even under these extreme conditions, the positioning error remains within the acceptable range, which indicates that the algorithm has a certain degree of robustness.

### 4.2 Performance comparison of different face recognition algorithms and comparison of different clustering methods

**Fig 11:**
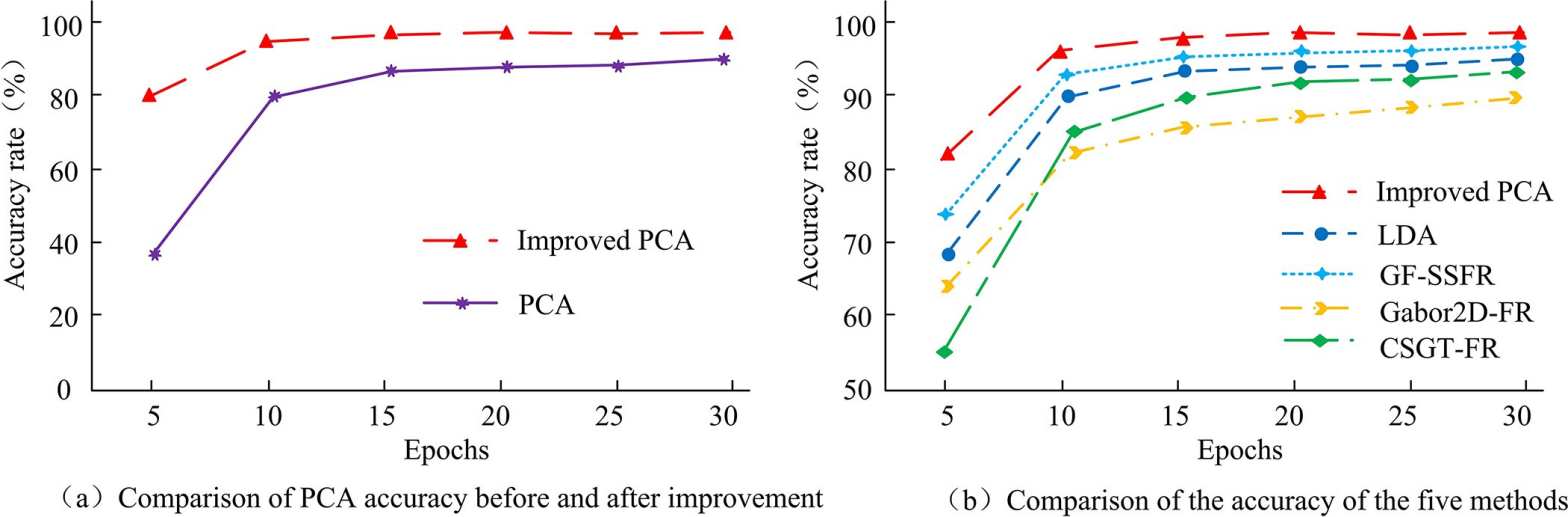
Comparison of accuracy of different methods.

To demonstrate the performance of Improved PCA more scientifically, this study compares it with Linear Discriminant Analysis (LDA) facial recognition algorithm, Subspace Facial Recognition Algorithm Combining Global and Local Features (GF-SSFR), Two-dimensional Gabor Wavelet Based Facial Recognition Algorithm (Gabor2D-FR), and Circular Symmetric Gabor Transform Facial Recognition Algorithm (CSGT-FR). The accuracy comparison is shown in Fig 11.

**Fig 12:**
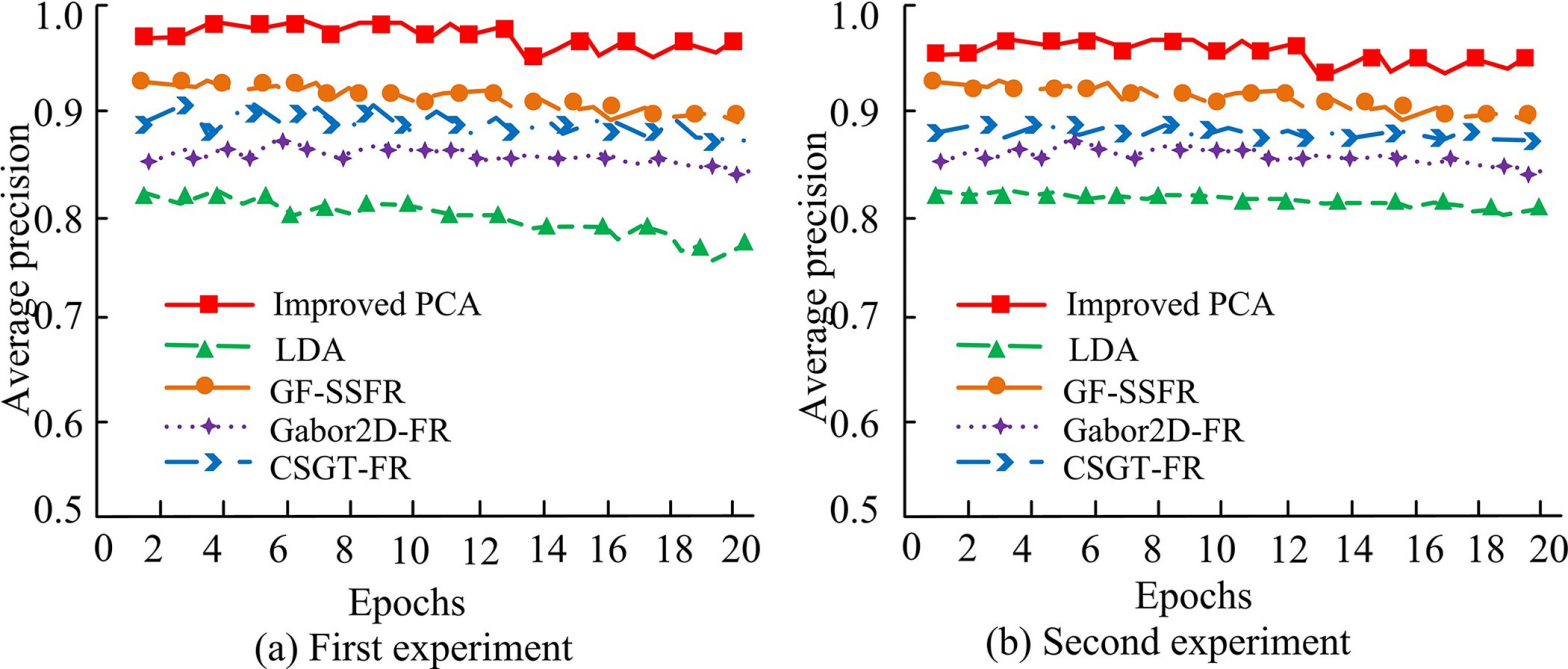
Evaluation accuracy between different models.

In Fig 11, the Improved PCA has the highest accuracy, reaching a maximum of 96%. PCA is a highly accurate facial recognition algorithm, surpassing other methods. This suggests that PCA is an excellent choice for facial recognition, especially after further research and improvement. The average accuracy of the five algorithms is compared, and the results are shown in Fig 12.

**Fig 13:**
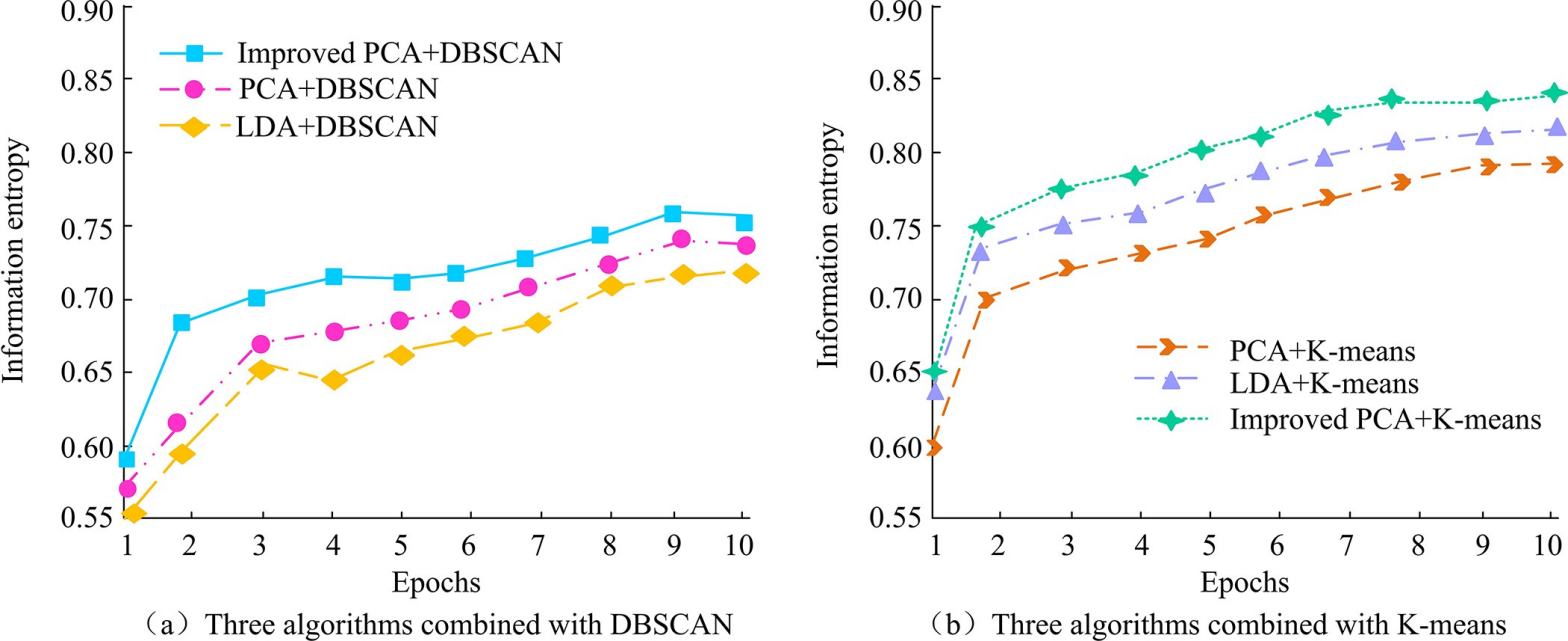
Comparison of information entropy.

In Fig 12, the average accuracy of LDA for facial recognition of 20 samples is 83%, GF-SSFR has an average accuracy of 90%, Gabor2D-FR possesses an average accuracy of 88%, and CSGT-FR has an average evaluation accuracy of 89%. The average evaluation accuracy of Improved PCA is 96%, which is higher than the average evaluation accuracy of all four models. The results show that the improved PCA algorithm has significant performance advantages in face recognition. This performance improvement is due to the improved PCA algorithm’s stronger ability in feature extraction and data dimensionality reduction, which more effectively captures key features of the face and reduces the interference of noise and irrelevant variables. The improved PCA face recognition algorithm has better recognition performance in the compared models, which provides an effective solution for face recognition technology. The Improved PCA and LDA algorithms are combined with the KA before and after improvement, and compared with the Densit-based Spatial Clustering of Applications with Noise (DBSCAN) algorithm. The comparison results of information entropy are shown in Fig 13.

**Fig 14:**
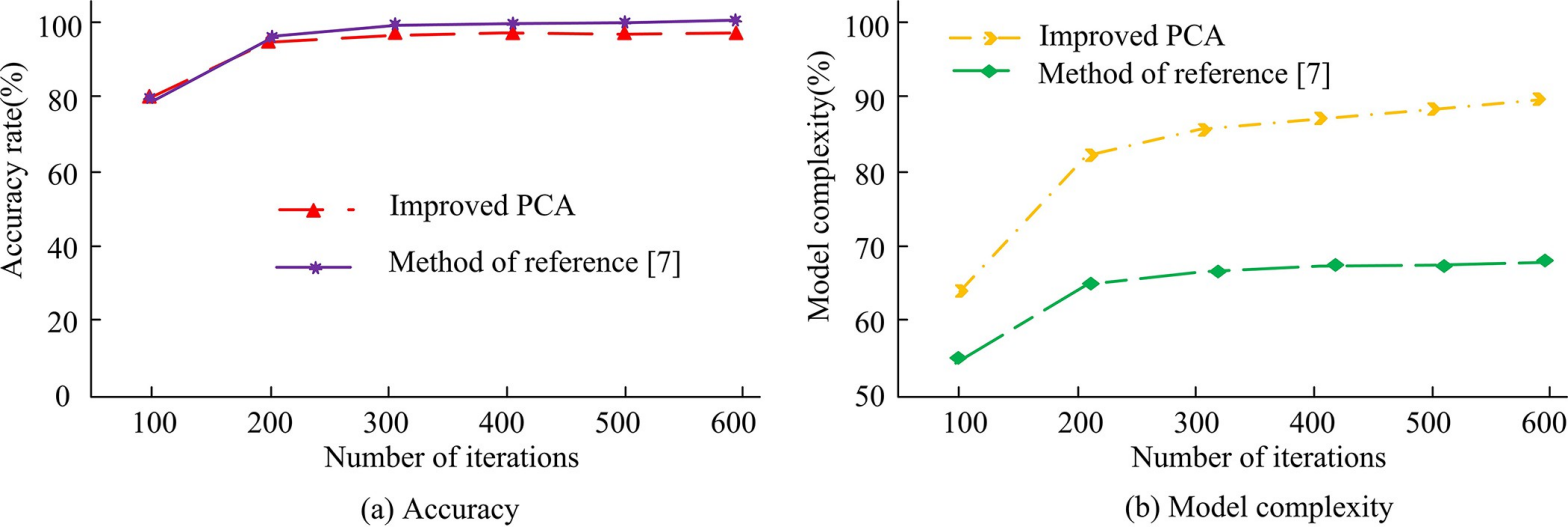
Comparison between the methods in reference [7] and the research methods.

In Fig 13, as the number of samples increases, the information entropy of each algorithm also increases. Information entropy is an index to measure the confounding degree of clustering effect. The higher the information entropy, the greater the uncertainty of clustering results, that is, the more chaotic the distribution of samples in clustering results. When algorithms are combined with DBSCAN, their information entropy is lower than that when they are combined with KA. The results demonstrate that, in the context of face recognition applications, the KA is capable of generating lower information entropy than the DBSCAN algorithm. This indicates that the KA is more effective than the DBSCAN algorithm in classifying samples into their corresponding categories during the clustering process, thereby reducing the uncertainty and clutter associated with classification. In particular, the improved PCA algorithm has the highest information entropy when combined with the KA. This indicates that the method employed in conjunction has the most optimal performance among all the evaluated algorithms, exhibiting the highest classification accuracy and the lowest degree of classification confounding. This combined method can effectively classify face samples into correct categories, reduce the possibility of misclassification, and improve the reliability of the face recognition system. The method in reference [7] is quite accurate, so it is compared with the method in this study to compare its accuracy and model complexity, as shown in Fig 14.

In Fig 14, the accuracy of the method in reference [7] is higher than that in this study, but the model complexity is correspondingly higher. In the method proposed in the reference [7], a convolutional neural network is used for pre-processing, feature extraction, and face recognition, and the Softmax operator is used as the classifier. The face recognition and classification of the input images are completed using the fully connected layer of the convolutional neural network, the classification function is learned using the fully connected layer of the network, and the output is classified using the Softmax layer. Such complex models may be able to better capture the inherent laws and patterns of the data, resulting in greater accuracy on the test data. Although the method in reference [7] shows advantages in terms of accuracy, higher model complexity may also bring some negative effects. More sophisticated models often demand greater computational resources and longer training and reasoning times, which can make deployment in real-world applications challenging or result in sub-optimal real-time performance. In addition, complex models are also more prone to over-fitting, that is, over-fitting training data and poor generalization ability. The statistical test of PCA face recognition system before and after improvement is shown in Table 3.

**Table 3:** Statistical test table of PCA face recognition system before and after improvement.

Inspection item	Sample size	Average recognition rate (%)	Standard deviation (%)	95% confidence interval	*p*-value (compared with before improvement)
Pre-modified PCA	100	85.0	5.2	83.4–86.6	< 0.001
Improved PCA	100	92.6	4.8	91.2–94.0	< 0.001

In Table 3, the standard deviation of PCA after improvement is 4.8%, which is slightly lower than 5.2% before improvement, indicating that the improved system performs more stable in different tests and the recognition rate fluctuates less. The confidence interval of the improved system is narrow, which indicates that the estimation of the improved recognition rate is more accurate and the reliability of the system performance is higher. The *p*-value before and after the improvement is less than 0.001, indicating that the improvement effect is statistically significant.

## 5. Conclusion

Face recognition technology has been extensively utilized in access control systems, which can improve the security and convenience of access control systems. This study utilized PCA in combination with the ASEF algorithm and KA to optimize the design and application of library FRACS. This study selected an appropriate dataset for data preprocessing, feature extraction, and real-time detection and recognition. The experiment showed that the accuracy and recall indicators of the improved PCA were relatively high, and the accuracy ultimately stabilized above 0.9, while the recall indicator also remained above 0.8. The recognition rate of the improved PCA library FRACS reached an average of 92.6%, and the recognition speed reached an average of 0.40s. The recognition rate of the improved PCA was about 10% higher than before. The recognition accuracy of improved PCA was higher than that of conventional PCA. The combination of improved PCA and KA resulted in the highest information entropy and the lowest level of clutter. The accuracy of improved PCA was higher than other algorithms, reaching a maximum of 96%. This study optimized the PCA facial recognition system, improved facial recognition rate, and improved facial recognition speed. The feasibility of optimizing stereo facial recognition has not been explored in the research, and it is hoped that further research will be conducted in the future.

## Supporting information

S1 Data set (DOC)

## References

[1] Mohsienuddin S. and Sabri M. S. "FACIAL RECOGNITION TECHNOLOGY." Social Science Research Network, vol. 7, no. 6, pp. 176–183, June, 2020, doi: 10.2139/ssrn.3622882

[2] Gidaris C. "The Problem with Regulating Facial Recognition Technology in a Digital Culture of Visibility." Canadian Journal of Communication, vol. 48, no. 1, pp. 124–141, June, 2023, doi: 10.3138/CJC.2022-0030

[3] Schuetz P. "Fly in the Face of Bias: Algorithmic Bias in Law Enforcement’s Facial Recognition Technology and the Need for an Adaptive Legal Framework." Law and Inequality: A Journal of Theory and Practice, vol. 39, no. 1, pp. 225–244, February, 2021, doi: 10.24926/25730037.391

[4] Lai X. J. and Rau P. L. P. "Has facial recognition technology been misused? A user perception model of facial recognition scenarios." Computers in Human Behavior, vol. 124, no. 1, pp. 106894.1–106894.13, November, 2021, doi: 10.1016/j.chb.2021.106894

[5] Fleet R. W. and Hine K. A. "Surprise, anticipation, sadness, and fear: A sentiment analysis of social media’s portrayal of police use of facial recognition technology." Policing: An International Journal of Police Strategies & Management, vol. 16, no. 4, pp. 630–647, December, 2022, doi: 10.1093/police/paab083

[6] Kar N. B., Nayak D. R., Babu K. S. and Zhang Y. D. "A hybrid feature descriptor with Jaya optimised least squares SVM for facial expression recognition." IET Image Processing, vol. 15, no. 7, pp. 1471–1483, July, 2021, doi: 10.1049/ipr2.12118

[7] Okokpujie K. and N John S. "Development of an Illumination Invariant Face Recognition System." International Journal of Advanced Technology in Computing Science Engineering, vol. 9, no. 5, pp. 9215–9220, March, 2021, doi: 10.30534/ijatcse/2020/331952020

[8] Ayo F. E., Mustapha A. M., Braimah J. A. and Aina D. A. "Geometric Analysis and YOLO Algorithm for Automatic Face Detection System in a Security Setting." Journal of Physics: Conference Series, vol. 32, no. 1, pp. 65–77, March, 2022.

[9] Alsawwaf M., Chaczko Z., Kulbacki M. and Sarathy N. "In Your Face: Person Identification Through Ratios and Distances Between Facial Features." International Journal of Computer Science and Applications, vol. 9, no. 2, pp. 187–202, October, 2021, doi: 10.1142/S2196888822500105

[10] Kapse A., Kamble T., Lohar A., Chaudhari S. and Puri D. "Face recognition Attendance system using HOG and CNN algorithm." International Conference on Advanced Computing and Communication, vol. 44, no. 1, pp. 03028–03035, May, 2022, doi: 10.1051/itmconf/20224403028

[11] Yeol H. S., Kim K. M. and Jik L. W. "Design and Implementation of Visitor Access Control System using Deep learning Face Recognition." Journal of Digital Convergence, vol. 19, no. 2, pp. 245–251, December, 2021, doi: 10.14400/JDC.2021.19.2.245

[12] Cai M., Shi Y., Liu J., Niyoyita J. P., Jahanshahi H. and Aly A. A. "DRKPCA-VBGMM: fault monitoring via dynamically-recursive kernel principal component analysis with variational Bayesian Gaussian mixture model." Journal of Intelligent Manufacturing, vol. 34, no. 6, pp. 2625–2653, March, 2023, doi: 10.1007/S10845-022-01937-W

[13] Li Z., Ying Y., Yang M., Zhao L. and Du W. "Monitoring and path optimization of catalytic reformer in a refinery: principal component analysis and A* algorithm application." Expert Systems with Applications, vol. 209, no. 12, pp. 118358.1–118358.11, December, 2022, doi: 10.1016/j.eswa.2022.118358

[14] Pascual H. and Yee X. C. "Least squares regression principal component analysis: A supervised dimensionality reduction method." Numerical Linear Algebra with Applications, vol. 29, no. 1, pp. 1–16, January, 2022, doi: 10.1002/nla.2411

[15] Manoilov V., Borodinov A. G., Petrov A., Zarutsky I. and Kurochkin V. "Machine learning algorithm for the construction of a nucleotide sequence in the nanofor sps sequencer using the principal component analysis." Nauchno-Priborostroitel’naya Gazeta, vol. 33, no. 2, pp. 35–36, February, 2023, doi: 10.18358/23122951_2023_33_2_35

[16] Kanuboyina V. S. N., Shankar T. and Penmetsa R. R. V. "Electroencephalography based human emotion state classification using principal component analysis and artificial neural network." Multidisciplinary Applications of Graphene Science, vol. 18, no. 3–4, pp. 263–278, March, 2022, doi: 10.3233/MGS-220333

[17] Esfe A. M. H. and Hajian M. "Prediction the dynamic viscosity of MWCNT-Al 2 O 3 (30:70)/ Oil 5W50 hybrid nano-lubricant using principal component analysis (PCA) with Artificial Neural Network (ANN)." Egyptian Informatics Journal, vol. 23, no. 3, pp. 427–436, September, 2022, doi: 10.1016/j.eij.2022.03.004

[18] Jiang X. and Sun H. W. "Learning performance of uncentered kernel-based principal component analysis." International Journal of Wavelets, Multiresolution and Information Processing, vol. 21, no. 3, pp. 2250059–2250075, March, 2023, doi: 10.1142/S021969132250059X

[19] Liu X. "Labor Market Resource Allocation Optimization Based on principal component analysis." Journal of Mathematics, vol. 2022, no. 2, pp. 1–11, February, 2022, doi: 10.1155/2022/1478013

[20] Kim J. H. and Kim E. K. "Face Recognition and Temperature Measurement Access Control System using Machine Learning." Journal of Korea Institute of Electronics and Communication Science, vol. 16, no. 1, pp. 197–202, February, 2021, doi: 10.13067/JKIECS.2021.16.1.19

[21] Jingade R. R. and Kunte R. S. "DOG-ADTCP: A new feature descriptor for protection of face identification system." Expert Systems with Applications, vol. 201, no. 1, pp. 117207–117208, September, 2022, doi: 10.1016/j.eswa.2022.117207

[22] Masood F., Masood J., Zahir H., Driss K., Mehmood N. and Farooq H. "Novel approach to evaluate classification algorithms and feature selection filter algorithms using medical data." Journal of Convergence for Computing, Electronics & Communications, vol. 2, no. 1, pp. 57–67, January, 2023, doi: 10.47852/bonviewJCCE2202238

