# Supplementary material for: Optimization design and application of library face recognition access control system based on improved PCA
Source: PLoS One. 2025 Jan 7;20(1):e0313415. doi: 10.1371/journal.pone.0313415 (PMC11706383; doi:10.1371/journal.pone.0313415)
Supplement: S1 Data set — (DOC) [file pone.0313415.s001.doc]

**The data in Figure 8**

| Epochs | Improved PCA | | | | PCA | | | |
| --- | --- | --- | --- | --- | --- | --- | --- | --- |
|  | Average fitness | Optimal fitness | Precision rate P | Recall rate R | Average fitness | Optimal fitness | Precision rate P | Recall rate R |
| 0 | 0.00 | 0.00 | 0.00 | 0.00 | 0.00 | 0.00 | 0.00 | 0.00 |
| 50 | 0.42 | 0.89 | 0.83 | 0.78 | 0.41 | 0.58 | 0.69 | 0.59 |
| 100 | 0.97 | 0.90 | 0.88 | 0.81 | 0.58 | 0.61 | 0.73 | 0.61 |
| 150 | 0.98 | 0.91 | 0.88 | 0.82 | 0.63 | 0.69 | 0.74 | 0.62 |
| 200 | 0.98 | 0.90 | 0.89 | 0.83 | 0.65 | 0.72 | 0.74 | 0.63 |
| 250 | 0.98 | 0.92 | 0.90 | 0.83 | 0.66 | 0.73 | 0.75 | 0.63 |
| 300 | 0.98 | 0.94 | 0.90 | 0.84 | 0.67 | 0.73 | 0.75 | 0.64 |
| 350 | 0.98 | 0.95 | 0.91 | 0.84 | 0.71 | 0.73 | 0.76 | 0.64 |
| 400 | 0.98 | 0.95 | 0.91 | 0.84 | 0.72 | 0.74 | 0.76 | 0.64 |

**The data in Figure 9**

| Feature face space dimension | Recognition rate (%) | | Recognition time (s) | |
| --- | --- | --- | --- | --- |
|  | PCA | Improved PCA | PCA | Improved PCA |
| 10 | 82 | 91 | 0.32 | 0.22 |
| 20 | 84 | 92 | 0.35 | 0.24 |
| 30 | 86 | 93 | 0.36 | 0.25 |
| 40 | 87 | 95 | 0.39 | 0.27 |
| 50 | 89 | 94 | 0.41 | 0.29 |

**The data in Figure 11**

| Epochs | Accuracy (%) | | | | | |
| --- | --- | --- | --- | --- | --- | --- |
|  | Improved PCA | PCA | LDA | GF-SSFR | Gabor2D-FR | CSGT-FR |
| 5 | 80.2 | 39.7 | 69.7 | 72.3 | 64.8 | 55.2 |
| 10 | 95.1 | 78.4 | 88.2 | 91.2 | 80.7 | 53.7 |
| 15 | 95.4 | 81.2 | 90.8 | 91.3 | 81.2 | 84.1 |
| 20 | 95.6 | 81.5 | 90.6 | 91.5 | 82.3 | 84.3 |
| 25 | 95.8 | 82.1 | 91.2 | 91.6 | 83.9 | 84.5 |
| 30 | 96.0 | 82.6 | 91.2 | 91.7 | 84.5 | 84.7 |

**The data in Figure 12**

| Epochs | Accuracy (%) | | | | | | | | | |
| --- | --- | --- | --- | --- | --- | --- | --- | --- | --- | --- |
|  | First experiment | | | | | Second experiment | | | | |
|  | Improved PCA | LDA | GF-SSFR | Gabor2D-FR | CSGT-FR | Improved PCA | LDA | GF-SSFR | Gabor2D-FR | CSGT-FR |
| 2 | 96.1 | 83.2 | 90.1 | 88.6 | 89.4 | 95.3 | 83.1 | 91.6 | 87.9 | 88.6 |
| 4 | 95.8 | 82.7 | 90.2 | 88.4 | 89.5 | 95.7 | 82.9 | 91.2 | 88.2 | 88.9 |
| 6 | 96.7 | 82.4 | 90.5 | 88.2 | 89.4 | 95.2 | 82.5 | 91.5 | 88.4 | 89.2 |
| 8 | 96.4 | 83.5 | 90.4 | 88.7 | 89.2 | 95.4 | 82.4 | 91.2 | 88.5 | 89.5 |
| 10 | 95.6 | 83.7 | 89.7 | 88.3 | 89.1 | 96.2 | 82.7 | 90.8 | 86.7 | 89.7 |
| 12 | 96.2 | 83.2 | 89.5 | 88.9 | 88.9 | 96.8 | 82.9 | 91.4 | 86.9 | 89.9 |
| 14 | 96.3 | 83.9 | 90.3 | 88.1 | 89.3 | 96.9 | 83.1 | 89.7 | 87.2 | 88.3 |
| 16 | 96.5 | 83.6 | 90.6 | 87.5 | 88.2 | 96.4 | 83.5 | 89.4 | 87.4 | 87.2 |
| 18 | 95.8 | 82.7 | 89.4 | 87.9 | 88.6 | 96.1 | 83.6 | 89.5 | 87.6 | 87.5 |
| 20 | 95.9 | 82.8 | 89.1 | 87.4 | 88.7 | 96.3 | 83.4 | 89.2 | 87.9 | 87.4 |

**The data in Figure 13**

| Epochs | Information entropy | | | | | |
| --- | --- | --- | --- | --- | --- | --- |
|  | Improved PCA+DBSCAN | PCA+DBSCAN | LDA+DBSCAN | PCA+K-means | LDA+K-means | Improved PCA+K-means |
| 0 | 0.00 | 0.00 | 0.00 | 0.00 | 0.00 | 0.00 |
| 1 | 0.58 | 0.57 | 0.55 | 0.60 | 0.64 | 0.65 |
| 2 | 0.67 | 0.62 | 0.59 | 0.69 | 0.69 | 0.75 |
| 3 | 0.71 | 0.66 | 0.64 | 0.73 | 0.72 | 0.76 |
| 4 | 0.70 | 0.67 | 0.62 | 0.74 | 0.73 | 0.77 |
| 5 | 0.72 | 0.68 | 0.65 | 0.75 | 0.75 | 0.79 |
| 6 | 0.72 | 0.68 | 0.66 | 0.75 | 0.77 | 0.80 |
| 7 | 0.74 | 0.69 | 0.67 | 0.77 | 0.78 | 0.83 |
| 8 | 0.75 | 0.69 | 0.69 | 0.78 | 0.79 | 0.83 |
| 9 | 0.75 | 0.72 | 0.70 | 0.79 | 0.80 | 0.84 |
| 10 | 0.74 | 0.74 | 0.71 | 0.79 | 0.80 | 0.85 |

**The data in Figure 14**

| Number of iterations | Accuracy rate (%) | | Model complexity (%) | |
| --- | --- | --- | --- | --- |
|  | Improved PCA | Method of reference [7] | Improved PCA | Method of reference [7] |
| 100 | 79.92 | 79.81 | 62.54 | 54.2 |
| 200 | 96.34 | 97.52 | 81.56 | 65.7 |
| 300 | 96.58 | 98.83 | 83.27 | 65.9 |
| 400 | 96.61 | 99.07 | 86.59 | 66.2 |
| 500 | 96.69 | 99.14 | 89.17 | 66.5 |
| 600 | 96.83 | 99.22 | 90.53 | 66.8 |
